# Supplementary figures and images for: Evidence of phenotypic plasticity along an altitudinal gradient in the dung beetle Onthophagus proteus
Source: PeerJ. 2021 Feb 24;9:e10798. doi: 10.7717/peerj.10798 (PMC7912602; doi:10.7717/peerj.10798)

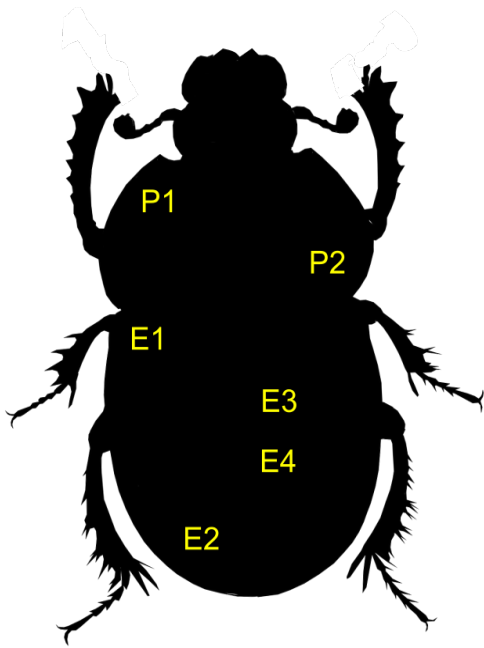

Supplement: Supplemental Information 1 [file peerj-09-10798-s001.pdf]

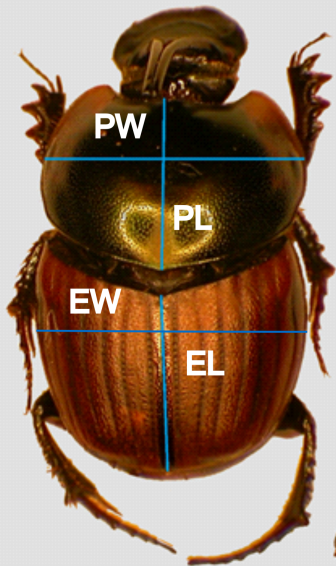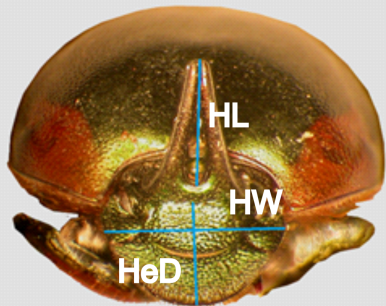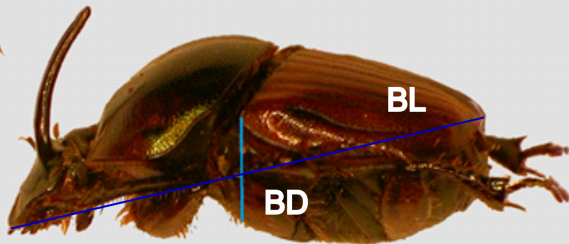

Supplement: Supplemental Information 2 [file peerj-09-10798-s002.pdf]
