# Supplementary material for: Evidence of phenotypic plasticity along an altitudinal gradient in the dung beetle Onthophagus proteus
Source: PeerJ. 2021 Feb 24;9:e10798. doi: 10.7717/peerj.10798 (PMC7912602; doi:10.7717/peerj.10798)
Supplement: Supplemental Information 3 [file peerj-09-10798-s003.docx]

| **Measurement** | **Measurement Description** | **Name** |
| --- | --- | --- |
| Head Width | Between the lateral basal margins of the genae | HW |
| Head Length | From the medial base to the medial apex of the head | HeD |
| Horn Length | From the medial base to the horn apex | HL |
| Pronotum Length | From the medial base to the medial apex of the pronotum | PL |
| Pronotum Width | Between the anterior lateral angles of the pronotum | PW |
| Elytra Length | From medial base to medial apex of elytra | EL |
| Elytra Width | Between anterior basal angles | EW |
| Body Depth | From the apical humeral callosity to the posterior of the mesothorax | BD |
| Body Length | From the medial base of the clypeus to the medial apex of the pygidium | BL |
|  |  |  |
|  |  |  |
