# Supplementary material for: Evidence of phenotypic plasticity along an altitudinal gradient in the dung beetle Onthophagus proteus
Source: PeerJ. 2021 Feb 24;9:e10798. doi: 10.7717/peerj.10798 (PMC7912602; doi:10.7717/peerj.10798)
Supplement: Supplemental Information 4 — The results of Kruskal Wallis test with Bonferroni corrections (H) used to compare morphological traits between individuals from different elevation bands and the results of Mann Whitney U-tests (w) with paired post-hoc tests to assess for morphological trait differences between sex. P values <0.05 are statistically significant. [file peerj-09-10798-s004.docx]

| Trait | Altitude Band | | | Sex | |
| --- | --- | --- | --- | --- | --- |
|  | H | df | *P* | *w* | *P* |
| Pronota length | 0.93 | 4 | 0.91 | 637 | 0.42 |
| Elytra length | 2.61 | 4 | 0.62 | 934 | 0.06 |
| Elytra width | 2.38 | 4 | 0.66 | 1176 | 0.74 |
| Abdomen depth | 2.55 | 4 | 0.63 | 1188 | 0.81 |
| Body length | 0.99 | 4 | 0.90 | 775 | 0.10 |
