# Supplementary material for: Evidence of phenotypic plasticity along an altitudinal gradient in the dung beetle Onthophagus proteus
Source: PeerJ. 2021 Feb 24;9:e10798. doi: 10.7717/peerj.10798 (PMC7912602; doi:10.7717/peerj.10798)
Supplement: Supplemental Information 5 [file peerj-09-10798-s005.docx]

| **Trait** | | |  |
| --- | --- | --- | --- |
| **Body Length** | W | *P* |  |
| BrGr | 168 | 0.26 |  |
| BrBr | 58 | 0.45 |  |
| BrBlk | 11 | 0.62 |  |
| BlkBlk | 3 | 0.57 |  |
| GrBlk | 14 | 0.99 |  |
| **Elytron Length** |  |  |  |
| BrGr | 171 | 0.29 |  |
| BrBr | 55 | 0.36 |  |
| BrBlk | 9 | 0.77 |  |
| BlkBlk | 14 | 0.90 |  |
| GrBlk | 5 | 0.99 |  |
|  | | | |
